# High prevalence of HIV infection among homeless and street-involved Aboriginal youth in a Canadian setting

**DOI:** 10.1186/1477-7517-5-35

**Published:** 2008-11-19

**Authors:** Brandon DL Marshall, Thomas Kerr, Chris Livingstone, Kathy Li, Julio SG Montaner, Evan Wood

**Affiliations:** 1 British Columbia Centre for Excellence in HIV/AIDS, St. Paul's Hospital, 608-1081 Burrard Street, Vancouver, BC, V6Z 1Y6, Canada; 2 School of Population and Public Health, University of British Columbia, 5804 Fairview Avenue, Vancouver, BC, V6T 1Z3, Canada; 3 Department of Medicine, University of British Columbia, St. Paul's Hospital, 608-1081 Burrard Street, Vancouver, BC, V6Z 1Y6, Canada; 4 Western Aboriginal Harm Reduction Society, 380 East Hastings Street, Vancouver, BC, V6A 1P4, Canada

## Abstract

Aboriginal people experience a disproportionate burden of HIV infection among the adult population in Canada; however, less is known regarding the prevalence and characteristics of HIV positivity among drug-using and street-involved Aboriginal youth. We examined HIV seroprevalence and risk factors among a cohort of 529 street-involved youth in Vancouver, Canada. At baseline, 15 (2.8%) were HIV positive, of whom 7 (46.7%) were Aboriginal. Aboriginal ethnicity was a significant correlate of HIV infection (odds ratio = 2.87, 95%CI: 1.02 – 8.09). Of the HIV positive participants, 2 (28.6%) Aboriginals and 6 (75.0%) non-Aboriginals reported injection drug use; furthermore, hepatitis C co-infection was significantly less common among Aboriginal participants (*p *= 0.041). These findings suggest that factors other than injection drug use may promote HIV transmission among street-involved Aboriginal youth, and provide further evidence that culturally appropriate and evidence-based interventions for HIV prevention among Aboriginal young people are urgently required.

## Background

Aboriginal populations in Canada are contending with a disproportionate burden of HIV infection [1]. Although only 3.3% of Canadians identify as American Indian, First Nations, Inuit, or Métis, Aboriginal people accounted for 18.8% of HIV test reports in 1998 and 27.3% in 2006 [1,2]. Within adult Aboriginal communities, injection drug use is considered to be one of the primary modes of HIV transmission, accounting for approximately 60% of new HIV infections [1]. Among injection drug using populations, Aboriginal ethnicity has also been shown to be an independent predictor of HIV seroconversion [3,4]. Elevated rates of HIV incidence have also been observed among young Aboriginal injection drug users [5,6]. Although the prevalence and risk factors for HIV infection among Aboriginal injection drug users have been relatively well-described, there exists little information on HIV infection among populations of street-involved Aboriginal youth with heterogeneous (i.e., injection and non-injection) drug-using characteristics and patterns. Since HIV infections typically occur at earlier ages among Aboriginal people as compared to the non-Aboriginal population [1], research examining the risk factors for HIV infection among this age group is of particular salience to public health programming and policy. We undertook this study to examine the prevalence and characteristics of HIV positive status among a cohort of street-involved youth in Vancouver.

## Methods

The At Risk Youth Study (ARYS) is a prospective cohort of drug-using and street-involved youth that has been described in detail previously [7]. Snowball sampling and extensive street-based outreach was conducted to recruit participants into the study. Eligibility criteria included: being between the age of 14 and 26, self-reported use of illicit drugs other than or in addition to marijuana in the past 30 days, and the provision of informed consent. The study has been approved by the University of British Columbia/Providence Health Care Research Ethics Board. We also sought to ensure that the research protocols were in accordance with the Canadian Institutes of Health Research *Guidelines for Health Research Involving Aboriginal People *[8].

All participants who completed a baseline survey between September, 2005 and October, 2006 were included in this analysis. At study entry, each participant completed an interviewer-administered questionnaire and provided blood samples for HIV and hepatitis C (HCV) serology. American Indian/Aboriginal ethnicity (yes vs. no) was defined as self-identified First Nations, Aboriginal, Inuit, or Métis origin. Other variables that were included in this analysis included age (<22 vs. ≥ 22), sex (female vs. male), Downtown Eastside (DTES) residency, homelessness, injection drug use, syringe sharing, history of incarceration, history of sex work, history of sexual abuse, ever engaging in anal intercourse, condom use (inconsistent vs. consistent), and for males, ever engaging in sex with men. As described previously [9], individuals were recorded as residents of the Downtown Eastside if they responded "DTES" to the question, "What local neighbourhoods or cities have you lived in during the past 6 months". Individuals classified as DTES residents may include those who are homeless and sleep or spend most of their time in the neighbourhood. To be consistent with previous studies, syringe sharing included lending or borrowing used syringes, and inconsistent condom use was defined as not always using a condom during vaginal and anal intercourse with all regular and casual partners [10,11].

Pearson's chi-square test was used to determine the factors associated with HIV positive status at baseline (Table 1). Fisher's exact test was used when one or more of the cell counts was less than or equal to five. Since we only observed 15 positive diagnoses, multivariate analysis was not conducted; however, the individual characteristics of each HIV positive participant were aggregated and are presented in Table 2.

**Table 1:** Factors associated with HIV seropositive status among a cohort of homeless and street-involved youth (*n *= 529)

Characteristic	HIV Positive n (%) n = 15	HIV Negative n (%) n = 514	Odds Ratio (95% CI)	*p*-value
Age				
< 22	2 (13.3)	265 (51.6)	0.14 (0.03 – 0.65)	0.003
≥ 22	13 (86.7)	249 (48.4)		
Sex				
Female	5 (33.3)	153 (29.8)	1.18 (0.40 – 3.51)	0.778
Male	10 (66.7)	361 (70.2)		
Aboriginal Ethnicity				
Yes	7 (46.7)	120 (23.3)	2.87 (1.02 – 8.09)	0.037
No	8 (53.3)	394 (76.7)		
DTES Residency^†^				
Yes	4 (26.7)	139 (27.0)	0.98 (0.31 – 3.13)	1.000
No	11 (73.3)	375 (73.0)		
Homeless^†^				
Yes	11 (73.3)	393 (76.5)	0.85 (0.26 – 2.71)	0.761
No	4 (26.7)	121 (23.5)		
Injection Drug Use^†^				
Yes	8 (53.3)	151 (29.4)	2.75 (0.98 – 7.73)	0.046
No	7 (46.7)	363 (70.6)		
Syringe Sharing^†^				
Yes	3 (20.0)	45 (8.8)	2.59 (0.70 – 9.56)	0.148
No	12 (80.0)	467 (91.2)		
Incarceration^‡^				
Yes	11 (73.3)	382 (74.3)	0.95 (0.30 – 3.04)	1.000
No	4 (26.7)	132 (25.7)		
Sex Work^‡^				
Yes	8 (53.3)	107 (20.8)	4.35 (1.54 – 12.26)	0.003
No	7 (46.7)	407 (79.2)		
Sexual Abuse^‡^				
Yes	6 (42.9)	132 (26.0)	2.13 (0.73 – 6.23)	0.271
No	8 (57.1)	375 (74.0)		
MSM^‡^				
Yes	2 (13.3)	33 (6.4)	2.24 (0.49 – 10.36)	0.261
No	13 (86.7)	481 (93.6)		
Anal Intercourse^‡^				
Yes	5 (33.3)	149 (29.0)	1.22 (0.41 – 3.64)	0.774
No	10 (66.7)	365 (71.0)		
Condom Use* ^†^				
Inconsistent	4 (57.1)	284 (69.6)	0.58 (0.13 – 2.65)	0.442
Consistent	3 (42.9)	124 (30.4)		

Note: † – refers to activities in the past 6 months; ‡ – refers to lifetime history; * – among sexually active participants

**Table 2:** Characteristics of HIV positive homeless and street-involved youth (*n *= 15).

Characteristic	Aboriginal n (%) n = 7	Non-Aboriginal n (%) n = 8
Age < 22	1 (14.3)	1 (12.5)
Female	3 (42.9)	2 (25.0)
DTES Residency^†^	3 (42.9)	1 (12.5)
Homeless^†^	5 (71.4)	6 (75.0)
Injected Drugs^†^	2 (28.6)	6 (75.0)
Shared Syringes^†^	0 (0.0)	3 (37.5)
Incarceration^‡^	7 (100.0)	5 (62.5)
Sex Work^‡^	4 (57.1)	4 (50.0)
Sexual Abuse^‡^	4 (57.1)	2 (25.0)
MSM^‡^	2 (28.6)	0 (0.0)
Anal Intercourse^‡^	2 (28.6)	3 (37.5)
Inconsistent Condom Use^†^	2 (28.6)	2 (25.0)
Hepatitis C Infection	1 (14.3)	6 (75.0)

Note: † – refers to activities in the past 6 months; ‡ – refers to lifetime history;

## Findings

A total of 529 participants completed a baseline survey and were eligible for this analysis. The median age of the sample was 22.0 (interquartile range: 19.9 – 23.9), 159 (30.1%) were female, 404 (76.4%) had been homeless in the past six months, and 221 (41.8%) reported ever injecting. In total, 127 (24.0%) participants self-identified as Aboriginal, American Indian, First Nations, Inuit, or Métis.

Of the entire sample, 15 (2.8%) tested positive for HIV, of whom 7 (46.7%) were of Aboriginal ethnicity. As shown in Table 1, Aboriginal ethnicity was associated with HIV infection (odds ratio [OR] = 2.87, 95%CI: 1.02 – 8.09), as was injection drug use (OR = 2.75, 95%CI: 0.98 – 7.73) and sex trade work (OR = 4.35, 95%CI: 1.54 – 12.26). Younger participants were less likely to be infected with HIV (OR = 0.14, 95%CI: 0.03 – 0.65).

Among the HIV positive individuals (Table 2), only 2 (28.6%) Aboriginal participants reported injecting drugs and none reported sharing syringes. HIV-infected Aboriginal youth were significantly less likely to be co-infected with HCV (Fisher's exact test *p*-value = 0.041).

## Discussion

Among a community-based sample of street-involved youth, Aboriginal participants were more than two and a half times more likely to be infected with HIV. The prevalence of HIV among Aboriginal youth in this sample was 5.5%, a proportion similar to that reported in a recent study of at-risk Aboriginal youth in two cities in British Columbia [12]. The prevalence of HIV among Aboriginal youth in this setting is also substantially higher than those that have been observed among street youth populations in Montréal (1.9%) and Toronto (2.2%) [13,14]. Furthermore, the fact that HIV-infected Aboriginal youth were less likely to report injection drug use and be co-infected with HCV suggests that unsafe sexual activity, sex work, and other unmeasured antecedent factors may be responsible for a significant proportion of infections. These findings are concerning and suggest that immediate and culturally appropriate policy and programmatic remedies are required to prevent further infections among Aboriginal youth and to provide increased resources to those individuals who are already infected.

Other factors that were associated with HIV positivity in bivariate analysis are similar to other studies of HIV infection among street-involved youth in Canada. For example, older age, history of injection drug use, and sex work were also all significant correlates of HIV infection among a cohort of street-involved youth in Montreal [14]. Of particular relevance to our setting is the high prevalence of incarceration observed among both HIV positive and negative participants – in fact, all seven HIV positive Aboriginal individuals also reported a history of incarceration. Given that incarceration has been associated with both HIV risk behaviours [15] and HIV incidence [16] in Vancouver, interventions to reduce street youths' exposure to correctional environments and the HIV-related harms associated with them are in urgent need of evaluation. Of further concern is that over half of HIV-infected Aboriginal participants reported experiencing sexual abuse, a finding which supports a recent study showing strong associations between sexual abuse and HIV risk behaviours among this population [17]. These results suggest that programs which aim to support HIV positive Aboriginal young people should recognize and address the lasting effects of historical trauma and cultural assimilation stemming from the Canadian residential school system on current levels of sexual abuse, substance use, and other HIV-related vulnerabilities.

Recently, the federal government of Canada announced that funding to community and regional HIV programs would be redirected towards the Canadian HIV Vaccine Initiative [18,19]. Although research funding for HIV vaccines is undoubtedly integral to long-term HIV strategies, the observed prevalence of HIV among Aboriginal youth observed in this and other studies supports statements made by the Assembly of First Nations that, relative to the size of the epidemic, HIV programs for Aboriginal programs are chronically under-funded and are in urgent need of further investment [20]. The Canadian Aboriginal AIDS Network has also argued that a serious lack of youth-specific HIV prevention programmes for Aboriginal youth exists across the country, and as such a national strategy on Aboriginal youth HIV/AIDS prevention is required [21]. Given these concerns, research and interventions that seek to identify effective strategies for addressing HIV infection and related vulnerabilities among Aboriginal young people should be a public health priority.

Our study is limited by its nonrandom sampling methodology that precludes generalization to the larger street-involved population in British Columbia. However, the sociodemographic characteristics of our sample are similar to those observed among other street youth studies in this setting [22,23]. Secondly, stigmatized behaviours such as injection drug use and syringe sharing may be underreported, particularly as the reliability and validity of self-report among samples of Aboriginal youth has been questioned by some authors [24]. However, a review of studies assessing the reliability and validity of self-reported drug use and HIV risk behaviours among injection drug users concluded that these measures are sufficiently valid [25]. It is also important to note that the prevalence of injection drug use and related behaviours reported in our study are similar to those from a recently published analysis of risk behaviours among Aboriginal youth who use drugs in Vancouver [12]. Even if socially desirable reporting were present in the data, we have no reason to believe that Aboriginal and non-Aboriginal participants would differ with respect to the likelihood of the underreporting of certain behaviours. Furthermore, it is noteworthy that biological evidence (i.e., hepatitis C serostatus) supports the self-reported data suggesting a higher proportion of sexually acquired HIV among Aboriginal participants. Finally, although we recognize that HIV vulnerability among Aboriginal populations is produced through a complex interplay of social, structural, and historical factors such as poverty, cultural oppression, and the multigenerational effects of the residential school system [6], we were unable to measure and characterize many of these effects.

In summary, we observed an alarmingly high prevalence of HIV infection among street-involved Aboriginal youth. Our findings demonstrate that urgent and culturally appropriate action is required to address the pervasive inequities that perpetuate marginalization and heightened vulnerability to HIV among Aboriginal young people in Canada.

## Competing interests

BM, TK, CL, KL, and EW declare that they have no competing interests. JM has received grants from, served as an ad hoc adviser to, or spoken at events sponsored by Abbott, Argos Therapeutics, Bioject Inc., Boehringer Ingelheim, BMS, Gilead Sciences, GlaxoSmithKline, Hoffmann-La Roche, Janssen-Ortho, Merck Frosst, Panacos, Pfizer, Schering, Serono Inc., TheraTechnologies, Tibotec (J&J), and Trimeris.

## Authors' contributions

EW had full access to all of the data and takes responsibility for the integrity of the results and the accuracy of the statistical analysis. BM conceived the study concept and design and was responsible for the composition of the manuscript.

The statistical analysis was conducted by KL and the interpretation of the results was performed by BM, CL, EW, JM and TK. The manuscript was edited and revised by BM, CL, EW, JM and TK. All authors read and approved the final manuscript.
